# This supplement is dedicated to the late Dr. Richard Warrington

**DOI:** 10.1186/s13223-024-00944-1

**Published:** 2025-01-27

**Authors:** Harold Kim, Anne K. Ellis, Wade Watson

**Affiliations:** 1 Division of Clinical Immunology and Allergy, Department of Medicine, McMaster University, Hamilton, Ontario Canada; 2 Division of Clinical Immunology and Allergy, Department of Medicine, Western University, London, Ontario Canada; 3 Division of Allergy and Immunology, Department of Medicine, Queen’s University, Kingston, ON Canada; 4 Division of Allergy, IWK Health Centre, Department of Pediatrics, Dalhousie University, Halifax, Nova Scotia Canada


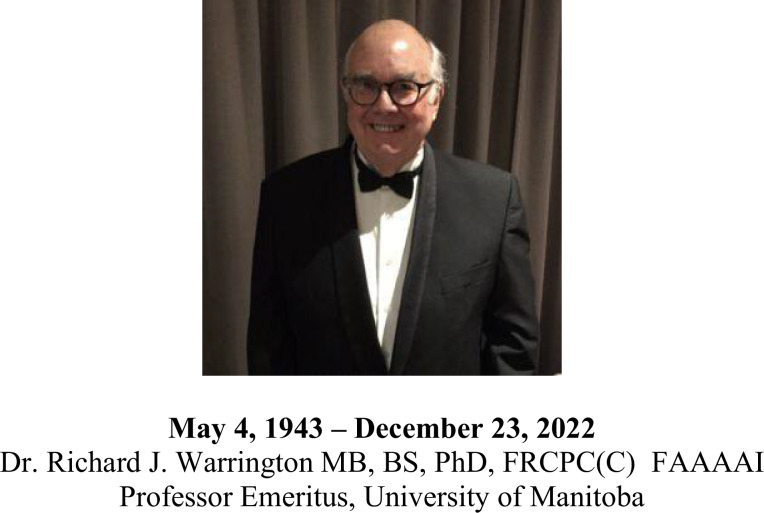


After a rich and full life dedicated to public service and research, Dr. Richard J. Warrington passed away unexpectedly on December 23rd, 2022, in Winnipeg, Manitoba, at the age of 79 years. The many people whom he touched in his life will remember his intelligence, humility, mentorship, kindness, humour, and empathy.

Dr. Warrington was a leading figure in the Canadian asthma and immunology community. He was Professor of Internal Medicine at the University of Manitoba (UM) and headed the section of Allergy and Clinical Immunology at UM for 39 years. His UM roles also included directing the Rheumatic Disease Unit Research Laboratory. He also served as Examiner, Chief Examiner and Vice-chair in Clinical Immunology and Allergy for the Royal College of Physicians and Surgeons of Canada.

Dr. Warrington was a research grant reviewer for 14 national councils and research foundations and a member of 18 national and international committees and boards. He conducted groundbreaking research, published more than 220 articles and abstracts in refereed journals, and had a stellar record of teaching and service.

Further to his experience and leadership roles, Dr. Warrington was appointed Editor-in-Chief of the *Allergy, Asthma & Clinical Immunology* journal in 2004 and remained in that position until his passing. He was President of the Canadian Society of Allergy and Clinical Immunology (CSACI) in 2008–2009, and subsequently was elected the first Honorary Member. He received numerous honours for his distinguished record of achievement and professional service, including the Jerry Dolovich Award for contributions in allergy and clinical immunology in Canada.

Dr. Warrington’s compassionate guidance and commitment to advancing the field of allergy and immunology have left an indelible mark on both his colleagues and the countless lives touched by his work. May his legacy and spirit of service to his patients and colleagues continue to inspire us all.

